# A method for quantifying pulmonary *Legionella pneumophila* infection in mouse lungs by flow cytometry

**DOI:** 10.1186/1756-0500-5-448

**Published:** 2012-08-20

**Authors:** Desmond Koon Yong Ang, Sze Ying Ong, Andrew Stephen Brown, Elizabeth Louise Hartland, Ian Richard van Driel

**Affiliations:** 1 Department of Biochemistry and Molecular Biology, Bio21 Molecular Science and Biotechnology Institute, The University of Melbourne, Parkville, VIC, 3010, Australia; 2 Department of Microbiology and Immunology, The University of Melbourne, Parkville, VIC, 3010, Australia

**Keywords:** *Legionella pneumophila*, Legionnaires’ disease, Neutrophil, Flow cytometry, Mouse infection model

## Abstract

**Background:**

Pulmonary load of *Legionella pneumophila* in mice is normally determined by counting serial dilutions of bacterial colony forming units (CFU) on agar plates. This process is often tedious and time consuming. We describe a novel, rapid and versatile flow cytometric method that detects bacteria phagocytosed by neutrophils.

**Findings:**

Mice were infected with *L. pneumophila* via intratracheal or intranasal administration. At various times after bacteria inoculation, mouse lungs were harvested and analysed concurrently for bacterial load by colony counting and flow cytometry analysis. The number of *L. pneumophila*-containing neutrophils correlated strongly with CFU obtained by bacteriological culture.

**Conclusions:**

This technique can be utilised to determine pulmonary bacterial load and may be used in conjunction with other flow cytometric based analyses of the resulting immune response.

## Findings

### Introduction

*Legionella pneumophila* is an opportunistic intracellular pathogen of humans and the major cause of Legionnaires’ disease worldwide. Infection of mice with *L. pneumophila* has been widely utilised to investigate the contribution of both host and pathogen derived factors to infection and the resulting immune response [1]. A critical technique in these investigations is the quantification of pulmonary bacteria in infected mice. This is usually performed by determining the number of bacterial colony forming units (CFU) through plating homogenised lung tissue on bacteriological agar [2]. Viable bacteria in the lung are inferred from this result. This process is tedious and time consuming due to the need for serial dilution of samples and slow growth of *L. pneumophila* on agar plates [3]. Also, using lung tissue to determine CFU limits its availability for other analyses such as the recruitment of various immune cells, determining the activation status of these cells and the production of cytokines. We describe a novel flow cytometry based technique for the enumeration of pulmonary bacteria in *L. pneumophila* infected mice. This technique relies on the fact that neutrophils traffic into the lungs and phagocytose bacteria during *L. pneumophila* infection [2,4]. Therefore, the enumeration of neutrophils that contain intracellular bacteria serves as a rapid and quantitative measure of pulmonary bacterial load and can be used in conjunction with other flow cytometric based analyses of the resulting immune response.

### Methods

All animal experimentation was approved by the University of Melbourne Animal Experimentation Ethics Committee. All mice were obtained and maintained in the Department of Microbiology and Immunology animal facility, The University of Melbourne. C57BL/6 mice have a more active flagella-sensing Naip5/Birc1e compared with A strain mice and thus are able to clear wild-type bacteria more effectively. Therefore, to achieve robust infection in C57BL/6 mice, an aflagellated Δ*flaA* mutant strain of bacteria was used [5,6]. To establish pulmonary infection, 6 to 10 week old A strain or C57BL/6 mice were anaesthetised and inoculated intratracheally with 2.5 × 10^6^ CFU of *L. pneumophila* strain 130b (ATCC BAA-74) or Δ*flaA* JR32 [7], respectively, as previously described [8,9]. Some mice were inoculated via intranasal inhalation of 2.5 × 10^6^ CFU of bacteria suspended in 50 μl of PBS. At 1, 2, 5 or 7 days after infection, mice were killed by CO_2_ asphyxiation and the lungs removed. One half of the lungs were prepared for CFU counting on agar plates as previously described [8,9]. The other half of the lungs were prepared for flow cytometry intracellular staining in the following manner: Each lung sample was finely minced using scissors and enzymatically digested in 3 ml of RPMI-1640 media (Gibco, Invitrogen) supplemented with 3% foetal calf serum, 0.1% DNAse (Sigma-Aldrich) and 0.1% collagenase type III (Worthington-Biochemical). Tissue digestion was achieved by constant and gentle pipetting for 20 min at room temperature. Undigested tissue was filtered out and red blood cells were removed using RBC Lysis Buffer (BD Biosciences) according to the manufacturer’s recommendations. The resulting cell suspension was labelled with PE-Cy7 conjugated anti-CD45 (Clone 30-F11, eBioscience), PE conjugated anti-Ly6G (Clone 1A8, BD Biosciences) and APC-eFluor780 conjugated anti-CD11b (Clone M1/70, eBioscience). The choice of anti-Ly6G antibody was critically important as antibody clones other than 1A8 cross react with Ly6C, a marker that is expressed on monocytes [10]. Cells were incubated for 20 min at 4°C, washed and permeabilised for intracellular staining using reagents and protocols supplied by eBioscience (Foxp3/Transcription factor staining buffer set. Cat. no. 00-5523-00). Briefly, cells were permeabilised with the eBioscience Fixation/Permeabilisation solution for 30 min at 4°C followed by one wash with the eBioscience Permeabilisation buffer. Cells were labelled with FITC-conjugated polyclonal rabbit anti-*Legionella pneumophila* antibody (ViroStat, Cat. no. 6053), diluted 1:1000 in the eBioscience Permeabilisation buffer, for 30 min at 4°C, washed and resuspended for flow cytometric analysis. A FITC-conjugated polyclonal rabbit IgG was used as an isotype staining control (eBioscience, Cat. no. 11-4614-80). 20,000 APC beads (BD Biosciences) were included into each sample to calculate the total number of cells for the whole lung based on a method adapted from [11]. Cells were analysed on a Becton Dickinson LSRFortessa flow cytometer using FACSDIVA software (BD Biosciences). Correlation and statistical significance (p) was determined by calculating the Spearman’s rank correlation coefficient (r_s_) using Prism software (GraphPad).

### Results and conclusion

**Figure 1:**
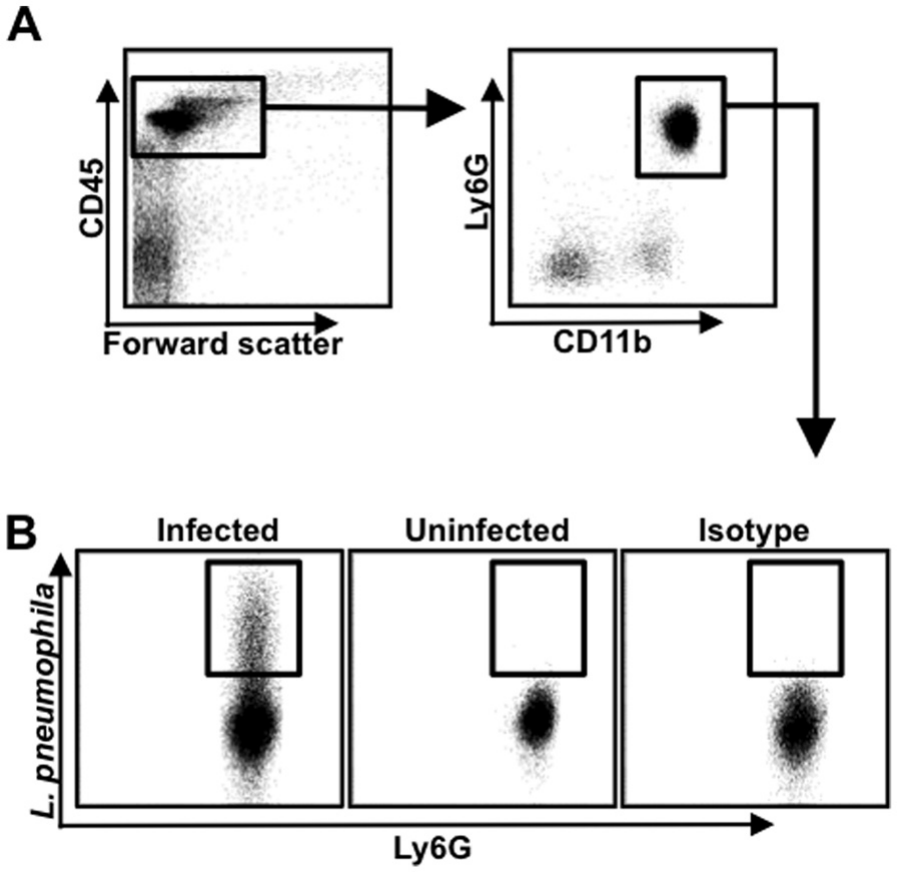
** Gating strategy for the identification of *L. pneumophila*-containing neutrophils.** Mice were infected with 2.5 × 10^6^ CFU of *L. pneumophila* per mouse. Uninfected mice received PBS. Lungs were harvested at various times after infection and analysed for the presence of neutrophils that contained intracellular bacteria. **A**. Representative plots showing the presence of CD45^+^CD11b^+^Ly6G^+^ neutrophils in the lungs of infected mice. **B**. Representative plots showing the staining of CD45^+^CD11b^+^Ly6G^+^ pulmonary neutrophils from infected or uninfected mice with an anti-*L. pneumophila* antibody (Infected and Uninfected) or from infected mice stained with an isotype-matched antibody with an irrelevant specificity (Isotype).

**Figure 2:**
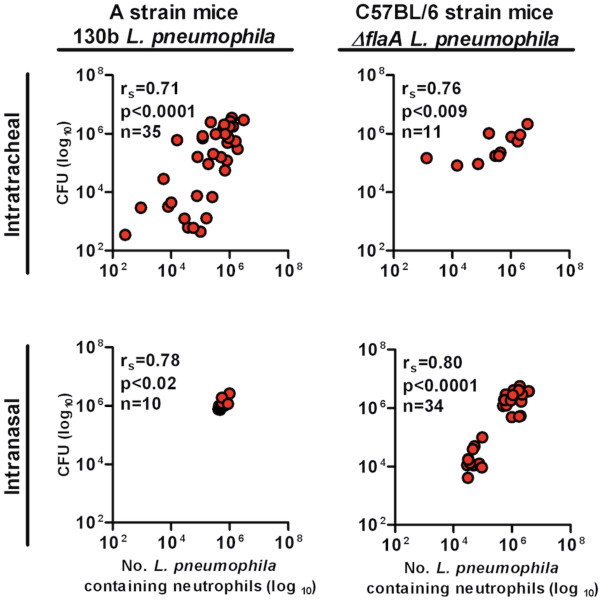
** The number of *L. pneumophila*-containing neutrophils correlates with data obtained by plating for CFU.** Mice were infected with 2.5 × 10^6^ CFU of *L. pneumophila* per mouse. Lungs were harvested at various times after infection and divided into two halves. One half was analysed for the presence of neutrophils that contained intracellular *L. pneumophila* by flow cytometry while the other was used to determine *L. pneumophila* CFU. Data from the two different methods were plotted and analysed for correlation by determining the Spearman’s rank correlation coefficient (r_s_) and statistical significance (p). Each circle represents the bacterial load in the whole lung for a single mouse. Each panel is a combination of 2 to 5 independent experiments with a range of 4 to 10 mice per experiment.

Figure 1 demonstrates the gating strategy used to identify neutrophils that contain intracellular bacteria. All leukocytes can be identified based on the expression of CD45. Neutrophils can then be identified based on expression of both Ly6G and CD11b (Figure 1A). The number of neutrophils that contain intracellular *L. pneumophila* was determined by the presence of FITC fluorescence relative to cells from uninfected mice and staining with an isotype-matched antibody with irrelevant specificity (Figure 1B). No staining of *L. pneumophila* was observed in neutrophils that were not permeabilised, indicating that the bacteria were located intracellularly (unpublished observation). The total number of neutrophils in the lung were then calculated based on the ratio of known APC beads added into each sample compared to the number of APC beads recorded by the flow cytometer [11]. To demonstrate that the number of *L. pneumophila*-containing neutrophils can serve as a quantitative measure of pulmonary bacterial load, these cell numbers were plotted against CFU data obtained from the same animals (Figure 2). Regardless of the method of bacterial inoculation or of the mouse/bacterial strain used, significant correlations were observed between the data generated by flow cytometry and those generated by CFU determination using the Spearman’s rank test, where a Spearman’s rank correlation coefficient (r_s_) approaching one represents increasing correlation between two variable datasets (Figure 2). 

Infection with *L. pneumophila* may lead to asymmetric distribution of bacteria in lung lobes. In these experiments, we observed statistically significant correlation and in most samples, the neutrophil value was very close to the CFU value suggesting that the impact of asymmetrical infection, if it did occur, was minimal. Nevertheless, to negate any such issues it would be wise to use all lung lobes for analysis if possible.

We have imaged pulmonary neutrophils containing *L. pneumophila* by fluorescent confocal microscopy and have found that the vast majority of neutrophils with anti-*Legionella* positive fluorescence contain bacterial fragments and not whole bacteria (unpublished observation). This is consistent with the primary function of neutrophils, which is to traffic to the site of infection to phagocytose and destroy bacteria. Based on this observation, we are unable to determine the number of bacteria contained within each neutrophil. Therefore, measurement of *L. pneumophila*-containing neutrophils does not directly quantitate live/cultivable bacteria. Nevertheless, data in Figure 2 clearly shows that the number of *L. pneumophila*-containing neutrophils is an accurate reflection of the severity of pulmonary infection as the CFU and the number of *L. pneumophila*-containing neutrophils demonstrated a statistically significant correlation. Therefore, the number of *L. pneumophila*-containing neutrophils can be used as an alternative method to compare the severity of *L. pneumophila* infection between individual mice in an experimental group. In a small number of samples with lower CFU values (< 10^5^ CFU/lung) we found that the number of *L. pneumophila*-containing neutrophils was greater than the CFU values. As most of the *L. pneumophila*-containing neutrophils contain bacterial fragments, we hypothesize that these may represent mice that are close to resolving the infection.

The biggest advantage of this flow cytometry based method is that a much shorter period is required to generate data. Typically, results can be obtained within 6 h after the harvest of lung tissue compared with up to 4 days required for bacterial colonies to grow on agar plates. Other advantages include the elimination of manual counting as the flow cytometer performs this function and that aseptic technique is no longer required to prevent contamination of agar plates by other bacterial genera. In addition, the enumeration can be performed alongside the analysis of other immune cells in infected mice. For instance, differences in the activation status, cytokine production or expression of functionally important markers between infected and uninfected immune cells can be determined. However, it is known that various host and pathogen factors can affect neutrophil recruitment and function in the lungs [12-15]. Therefore, validation of the flow cytometry method for bacterial load determination in other experimental models of *L. pneumophila* infection must be considered.

## Conclusion

In conclusion, we have developed a robust and reproducible flow cytometry based method for the quantification of pulmonary bacteria in the experimental mouse model of *L. pneumophila* infection. This method offers significant advantages over traditional CFU determination and may be adapted to other intracellular pathogens.

## Abbreviations

APC: Allophycocyanin; CFU: Colony forming units; FITC: Fluorescein isothiocyanate; PE: Phycoerythrin.

## Competing interest

The authors declare that they have no competing interests.

## Authors’ contributions

DKYA, SYO and ASB designed research, performed research, collected data, analysed and interpreted data, performed statistical analysis and wrote the manuscript. ELH, IRVD designed research, analysed and interpreted data and wrote/edited the manuscript. All authors have read and approved the final manuscript.
